# Smart Sensing Using Electromagnetic Waves for Inspection of Defects in Rock Bolts

**DOI:** 10.3390/s20102821

**Published:** 2020-05-15

**Authors:** Jung-Doung Yu, Jong-Sub Lee

**Affiliations:** 1 Department of Civil and Environmental Engineering, Stanford University, Stanford, CA 94305, USA; jungdoung@gmail.com; 2 School of Civil, Environmental, and Architectural Engineering, Korea University, Seoul 136-701, Korea

**Keywords:** defect, electromagnetic wave, nondestructive testing method, rock bolt, smart sensing

## Abstract

The stability of tunnels and rock slopes is adversely affected by defects in rock bolts. This study investigates the suitability of the smart sensing method using electromagnetic waves for inspecting defects in rock bolts. Experiments were performed with one fully grouted and eight defective rock bolts, out of which five have non-grouted parts at the ends with different non-grouted ratios, and three have different types of voids. Electromagnetic waves were generated and detected using a time domain reflectometer by configuring two-conductor transmission lines in the rock bolts. Results show that electromagnetic waves are reflected both at defects and ends of rock bolts. The electromagnetic wave velocity increases with an increase in the non-grouted ratio and decreases when rock bolts are embedded in a concrete block simulating rock mass. The estimated locations of defects found by electromagnetic waves are in good agreement with actual defect locations. This study demonstrates that smart sensing using electromagnetic waves is an effective method for inspecting and determining defect locations and the non-grouted ratio of rock bolts.

## 1. Introduction

Rock bolts are widely used as permanent or temporary support systems for stabilizing rock slopes and tunnels. Rock bolts are employed to bind weak rocks, such as fractured, discontinued, laminated, and jointed rocks, to strong rocks [1]. Thus, rock bolts reinforce unstable rock masses by preventing rock movements due to a sagging rock mass and rock separation [2]. A typical rock bolt installation procedure occurs in the following order: first, rock masses are drilled to make holes, next rebars are inserted into holes and finally, holes are filled with grout material to bond rebars to the surrounding rock mass. Various defects can be generated in rock bolts during construction. For example, grout materials occasionally flow out due to gravity, resulting in a non-grouted part at the end of the rock bolt. Furthermore, the collapse of the wall, ground water flow, insufficient insertion depth of tremie pipe, improper withdrawal rate of tremie pipe, and poor consistency of the grout mixture, grout flowing into voids of a fractured rock mass may adversely affect the grout quality and create voids in the grout [3,4,5,6,7]. Lee et al. [8] reported that the defect occurrence rate in tunnel construction is 15.8%. These defects weaken the bond strength between rock bolts and the surrounding rock, which depends on the chemical adhesion and friction after slip. Shear and tensile loads caused by rock movements are transferred to the grout, followed by the transfer of loads from grout to rebars [9]. Because the load transfer capacity of the rock bolt is dictated by the bond strength, defects in rock bolts should be monitored to guarantee the stability of rock slopes and tunnels.

The pull-out test, proposed by Barry et al. [10], is the most common method employed to inspect rock bolts today. Therein, the tensile capacity of rock bolts is estimated, which depends on the bond strength between rock bolts and surrounding rocks. However, the pull-out test can cause rock bolt deformation and/or destroy surrounding rocks, as the pull-out load is applied directly on the rock bolt. Furthermore, the pull-out test is known to be both cost and time-consuming [11,12], and it does not provide defect information. Buys et al. [13] have expressed the need to develop a nondestructive testing (NDT) method for inspecting rock bolt conditions to overcome the shortcomings of the conventional pull-out test. In the past twenty years, different NDT methods using smart sensors have been applied to inspect defects in rock bolts [14]. Among them, smart sensors for measuring ultrasonic guided waves have been the most widely utilized. Beard et al. [15] measured ultrasonic guided waves in rock bolts using an ultrasonic transducer. In their study, the ultrasonic guided wave arrival time and dispersion curves were analyzed to accurately determine the defect location and rock bolt length. Madenga et al. [16] measured ultrasonic guided waves transmitted from the end of the rock bolt using piezo-electric transducers and demonstrated that the velocity of ultrasonic guided waves is dependent on the grouted length of rock bolt, grout curing time, and excitation frequency. Han et al. [17] used a piezo disk element and acoustic emission (AE) sensor to generate and receive ultrasonic guided waves, respectively. In their study, the relationship between the propagation velocity and grouted ratio were suggested. Ultrasonic guided waves are dispersive and contain numerous high frequency components, making their arrival time in the rock bolt difficult to determine [18]. Previously, wavelet transforms were employed to accurately determine the arrival time of ultrasonic guided waves in rock bolts [18,19]. Lee et al. [18] performed an experimental study on ultrasonic guided waves in defective rock bolts. The guided waves were generated and detected using the piezo disk element and AE sensor, respectively. Ultrasonic guided waves transmitted from the end of the rock bolt were analyzed using the wavelet transform to estimate their energy and phase velocities in defective rock bolts. Stepinski and Matsson [20] measured ultrasonic guided waves within 100 kHz using a piezoelectric (PZT) element and analyzed their compressional and flexural modes in rock bolts. Rucka and Zima [21] installed a PZT plate actuator and laser vibrometer at the same end of the defective rock bolt for the generation and detection of ultrasonic guided waves, respectively. In their study, the attenuation coefficient of ultrasonic guided waves in the defective rock bolt was estimated to assess the energy loss in terms of the grouted length of the defective rock bolt. Yu et al. [22] performed experimental and numerical studies on ultrasonic guided waves in defective rock bolts. They investigated the relationship between the group velocity of ultrasonic guided waves and the grouted ratio of the defective rock bolt. Further, the velocity of ultrasonic guided waves in the rock bolt was found to be affected by the diameter and elastic modulus of grout materials. Bačić et al. [23] measured ultrasonic guided waves in rock bolts using an accelerometer. In their study, the relationship between the grouting quality and natural frequency of rock bolts was investigated.

Previous studies employing ultrasonic guided waves demonstrated significant achievements in the rock bolt inspection using smart sensors based on piezoelectric materials. However, a major limitation in the detection of an ultrasonic guided wave is that the propagation energy tends to leak from the rock bolt into the surrounding materials [24]. Moreover, smart sensors for detecting ultrasonic guided waves are expensive as high-priced electronics such as wave generators, signal conditioners and amplifiers are needed to produce and measure high quality signals. Furthermore, professional skills are required for the analysis of ultrasonic guided waves. Therefore, a novel sensing method allowing simple and facile signal analysis at low cost is required to inspect rock bolt defects. Recently, electromagnetic waves have been widely used for detecting defects in steel tendons or metals. Yu et al. [25] configured a single transmission in soil nails and investigated the travel time and velocity of electromagnetic waves in soils nails to evaluate the installed length of soil nails. Lee et al. [26] evaluated the grouted ratio of soil nails using electromagnetic waves by employing two of them as electrical conductors. Marindra and Tian [27] used a chipless RFID sensor tag for metal crack detection and characterization. In their study, variation in the resonant frequency of electromagnetic waves were investigated at different crack widths. Lee et al. [28] configured transmission lines with the reinforcement cage and estimated necking defect locations using the velocity of electromagnetic waves.

This study introduces a smart sensing method using electromagnetic waves to inspect defects in rock bolts, coupled with a simple, facile and low cost signal analysis procedure. Experimental studies were conducted on a fully grouted rock bolt and defective rock bolts. A waveguide system was established by configuring a two-conductor transmission line composed of a rebar and an electrical wire. Electromagnetic waves were generated on the transmission line using a time domain reflectometer (TDR) and detected using the same device. The presence of a defect was determined based on the reflection characteristics of electromagnetic waves. The non-grouted ratio of the rock bolt was evaluated by analyzing the round-trip time and propagation velocity of electromagnetic waves. The remainder of paper presents of the following sections: theoretical background, smart sensing system, waveform interpretation, experimental program, analyses and discussion, and summary and conclusions.

## 2. Theoretical Background

### 2.1. Transmission Line

**Figure 1:**
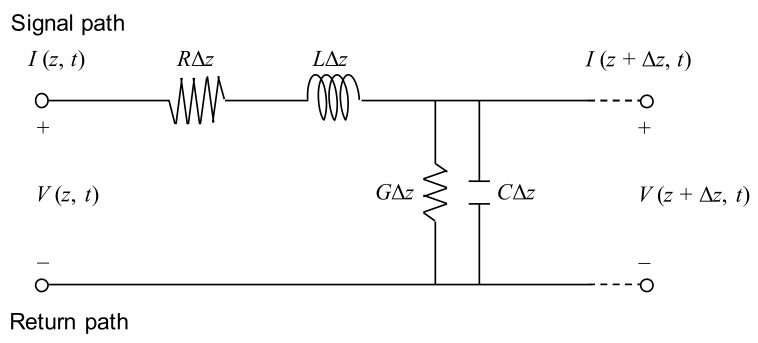
Circuit model of differential length Δ*z* of a two-conductor transmission line.

A transmission line is an electric circuit composed of two or more parallel conductors that transport an electrical signal from one point to another. Figure 1 shows an ideal circuit model of a two-conductor transmission line, which constitutes a pair of parallel conductors with transmission parameters *R*, *L*, *G*, and *C*, which represent the resistance per unit length in Ω/m, inductance per unit length in H/m, conductance per unit length in S/m, and capacitance per unit length in F/m, respectively. The propagation of electromagnetic waves along the transmission line depends on these parameters. The resistance is the series element of the transmission line, and it is dependent on the physical characteristics of the conductor at a given temperature. The resistance is the major cause behind electrical energy loss. The series inductance is an electrical property opposing a change in the current. It is expressed as the ratio of the number of magnetic flux lines to the current through the conductor. The inductance is dependent on the spacing, size, transposition, material, and arrangement of the conductor. The capacitance, which is a shunt element of the transmission line, is a measure of the capacity to store charges between conductors. It is dependent on the conductor size, spacing between conductors, and the dielectric constant of the material between conductors. The shunt conductance results from leakage currents and is expressed as the inverse of the resistance. A dielectric material between conductors affects electrical energy losses in the transmission line. In this study, the transmission line was configured by installing an electrical wire alongside the rebar, which is a conductive material made of a steel.

### 2.2. Electromagnetic Waves in Transmission Line

In a conventional two-conductor transmission line, a wave propagates in the transverse electromagnetic (TEM) mode; the electric and magnetic fields around conductors are both perpendicular to the direction of wave. Hence, the two-conductor transmission line supports the TEM wave. The electric and magnetic fields are uniquely related to voltage and current, respectively, as follows [29,30]:

(1) V=−∫E⋅dl

(2) I=∮H⋅dl

where *V* and *I* depict the voltage in volts and current in amperes, respectively; **E** and **H** represent the electric and magnetic field intensities, respectively; ***l*** is the conductor length. Given that *V* and *I* are expressed in terms of **E** and **H**, respectively, transmission line problems are solved with *V* and *I* instead of **E** and **H** for a simple and convenient calculation. Transmission line equations are obtained by applying Kirchhoff’s voltage and current laws [30]:

(3) $$V(z,t)=R\Delta zI(z,t)+L\Delta z\frac{\partial I(z,t)}{\partial t}+V(z+\Delta z,t)$$

(4) $$I(z,t)=I(z+\Delta z,t)-G\Delta zV(z+\Delta z,t)+C\Delta z\frac{\partial V(z+\Delta z,t)}{\partial t}$$

where *z* and *t* represent the time and length, respectively. Taking the limits of Equations (3) and (4) as Δ*x*→0 leads to:

(5) $$-\frac{\partial V(z,t)}{\partial z}=RI(z,t)+L\frac{\partial I(z,t)}{\partial t}$$

and:

(6) $$-\frac{\partial V(z,t)}{\partial z}=RI(z,t)+L\frac{\partial I(z,t)}{\partial t}$$

Assuming harmonic time dependence, Equations (5) and (6) become:

(7) $$-\frac{dV_{s}}{dz}=(R+j\omega L)I_{s}$$

and:

(8) $$-\frac{dI_{s}}{dz}=(G+j\omega C)V_{s}$$

respectively, where *V_s_* and *I_s_* are the phasor forms of *V* and *I*, respectively; *j* and *ω* are the imaginary number and angular frequency, respectively. The coupled *V_s_* and *I_s_* in Equations (7) and (8), respectively, are separated by taking the second derivative of *V_s_* in Equation (7) and employing Equation (8) as follows:

(9) $$-\frac{d^{2}V_{s}}{dz^{2}}-\gamma^{2}V_{s}=0$$

By taking the second derivative of *I_s_* in Equation (8) and employing Equation (7), the following equation is obtained:

(10) $$\frac{d^{2}I_{s}}{dz^{2}}-\gamma^{2}I_{s}=0$$

where:

(11) $$\gamma =\alpha +j\beta =\pm \sqrt{\left(R+j\omega L)(G+j\omega C\right)}$$

Equations (9) and (10) are wave equations for the voltage and current, respectively. *γ* is therefore a propagation constant (in units per meter), *α* is the attenuation constant (in nepers per meter), and *β* is the phase constant in radians per meter). The solutions of the linear homogeneous differential Equations (9) and (10) are expressed as:

(12) $$V_{s}(z)=V_{0}^{+}e^{-\gamma z}+V_{0}^{-}e^{\gamma z}$$

and:

(13) $$I_{s}(z)=I_{0}^{+}e^{-\gamma z}+I_{0}^{-}e^{\gamma z}$$

where *V*_0_^+^, *V*_0_^−^, *I*_0_^+^, and *I*_0_^−^ are wave amplitudes and the + and – signs denote the wave propagation in the +*z* and −*z* direction, respectively. The ratio of the amplitudes of the voltage wave to the current wave is the characteristic impedance (*Z*_0_):

(14) $$Z_{0}=\frac{V_{0}^{+}}{I_{0}^{+}}=-\frac{V_{0}^{-}}{I_{0}^{-}}=\sqrt{\frac{R+j\omega L}{G+j\omega C}}$$

According to the electromagnetic wave theory, the propagation velocity (*v_p_*) of the electromagnetic wave is given as [31]:

(15) $$v_{p}≅\frac{v_{c}}{\sqrt{\varepsilon_{r}}}$$

where *v_c_* depicts the velocity of electromagnetic waves in vacuum (3 × 10^8^ m/s). *ε_r_* is the relative permittivity, also referred to as the dielectric constant, which depicts the ratio of the permittivity of a material (*ε*) to the permittivity of vacuum (*ε*_0_).

If the electromagnetic waves propagating along a transmission line encounter an impedance discontinuity, they reflect and propagate in the opposite direction. The magnitude of the reflected electromagnetic waves is expressed as the reflection coefficient (*Γ*) [32]:

(16) $$\Gamma =\frac{V_{r}}{V_{i}}=\frac{I_{r}}{I_{i}}=\frac{-Z_{0}+Z_{1}}{Z_{0}+Z_{1}}$$

where *V_r_* and *V_i_* denote voltages of reflected and incident electromagnetic waves, respectively; *I_r_* and *I_i_* denote currents of reflected and incident electromagnetic waves, respectively; *Z*_0_ is the characteristic impedance (or electrical impedance of the medium 0), and *Z*_1_ is the electrical impedance of medium 1. The reflected signal appears with the same sign when *Z*_1_ > *Z*_0_, whereas the reflected signal appears with the opposite sign when *Z*_1_ < *Z*_0_.

The electrical impedance (*Z*) of the medium under investigation is related to the relative permittivity (*ε_r_*) [30]:

(17) $$Z=\sqrt{\frac{\mu_{0}}{\varepsilon_{0}\varepsilon_{r}}}$$

where *μ*_0_ is the magnetic permeability in vacuum. Assuming that *μ*_0_ and *ε*_0_ are constant, *Z* is inversely proportional to *ε_r_*. If the *ε_r_* of medium 0 is smaller than that of medium 1, *Z*_1_ will be larger than *Z*_0_, and the reflected signal will appear with the same sign. In contrast, the reflected signal will appear with the opposite sign if *ε_r_* of medium 0 is larger than that of medium 1, as *Z*_1_ is smaller than *Z*_0_.

## 3. Smart Sensing System

### 3.1. Configuration of Transmission Line

**Figure 2:**
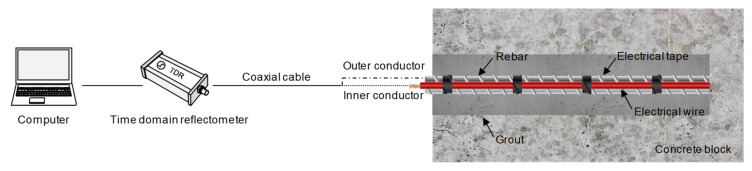
Smart sensing system for measuring electromagnetic waves in rock bolt.

Most rock bolts are made of steel rebars, which are a conductive material that allows the flow of electrical current. In this study, a two-conductor transmission line was configured with a rebar and an electrical wire, as shown in Figure 2. The SD 300 deformed steel rebar employed in this study has a diameter and length of 25.4 mm and 3.01 m, respectively. The two-conductor transmission line was configured by installing the electrical wire alongside the rebar. Electrical tapes were used to fix the electrical wire to the rebar at intervals of 0.2 m. The electrical wire is a single-conductor electrical wire composed of one piece of metallic conductor surrounded by plastic insulation. The metallic conductor was made of a flexible stranded annealed copper with a diameter of 2.1 mm. The plastic insulation constituted nonconductive material made of polyvinyl chloride (PVC) with a thickness of 0.8 mm. The velocity of electromagnetic waves in the rebar, where the transmission line was configured, was 2.649 × 10^8^ m/s, which is approximately 88.3% of the velocity of electromagnetic waves in vacuum (3 × 10^8^ m/s).

### 3.2. Generation and Detection of Electromagnetic Waves

**Figure 3:**
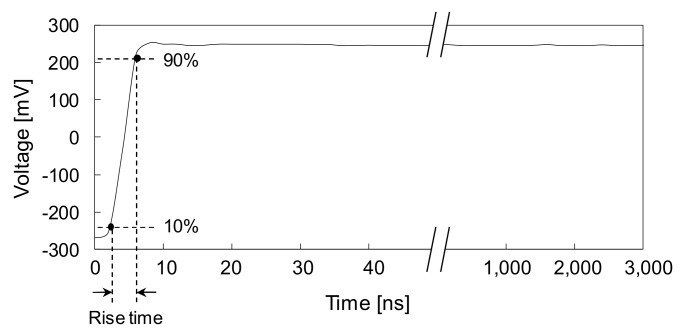
Step input signal generated by TDR.

A diagram of the smart sensing system for measuring electromagnetic waves in the rock bolt is illustrated in Figure 2. The generation and detection of electromagnetic waves were accomplished by means of a TDR. The TDR (HL1101, Hyperlabs, Beaverton, OR, USA) generated a step signal with an amplitude of ±250 mV and a width of 3 μs, as shown in Figure 3. The rise time of the input step signal was 200 ps between 10% and 90% of the maximum amplitude, corresponding to a frequency bandwidth of 1.75 GHz. The temporal resolution of the TDR was 10.2 ps. The TDR was connected to the transmission line configured in the rock bolt using a coaxial cable (RG-58A/U) with a characteristic impedance of 50 Ω. The electrical wire was set up as a path to transfer signals by connecting to the inner conductor of the coaxial cable. The rebar was set as the return path for receiving signals by connecting to the outer conductor of the coaxial cable. The electromagnetic waves reflected at discontinuities, such as defects, within and at the end of the rock bolt were finally detected by the TDR and recorded by a computer. In this study, 150 signals were stacked to improve the signal-to-noise ratio, and the total number of data points for each signal was set at 1024.

## 4. Waveform Interpretation

### 4.1. Signal Distortion

Signal distortion occasionally occurs owing to attenuation and dispersion of electromagnetic waves [33]. The attenuation of electromagnetic waves is attributed to the energy loss of the electromagnetic waves, which occurs when electromagnetic waves propagate in a lossy medium that absorbs significant energy. The energy of the signal is generally defined as a function of varying amplitude through time. Hence, it is estimated by the area under the signal envelope [34]. The attenuation reduces the amplitude of the signal without altering the pulse width (i.e., the energy is reduced while the pulse width is constant). The pulse width is generally defined as the time difference between the rising and falling edges of the pulse at points where the amplitude is 50% of the peak amplitude.

**Figure 4:**
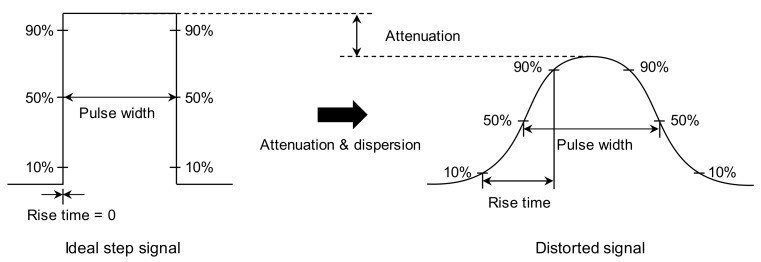
Signal distortion in lossy medium.

The electromagnetic waves traveling in a transmission line comprise various frequencies, and their velocity can be changed by altering the frequency. This phenomenon is referred to as signal dispersion. The dispersion therefore occurs regardless of whether the medium is lossy or not. The dispersion increases the velocity in higher-frequency components, resulting in a decrease in the pulse width and in an increase in the amplitude (i.e., the pulse width is reduced, while the energy remains constant). In contrast, the dispersion decreases the velocity in the lower-frequency components (i.e., the pulse width is increased, while the energy remains constant) [33,35]. Signal distortion generally occurs due to both attenuation and dispersion. As electromagnetic waves propagate through lossy medium, the higher-frequency signal components are attenuated, whereas the lower-frequency signal components remain constant [33]. Thus, the bandwidth of the signal decreases, whereas its rise time increases. Finally, the signal energy is reduced with increasing pulse width, as shown in Figure 4.

### 4.2. Determination of Travel Time

The round-trip travel time of reflected electromagnetic waves is generally determined by identifying inflection points of measured signals. The tangent and sloped line methods are the ones most commonly employed to identify signal inflection points [36,37,38]. The tangent method exhibits the best accuracy in determining an intersection between a horizontally flat and a sloped line [39,40]. The sloped line method was reported as suitable for determining an intersection between two sloped lines [41,42].

**Figure 5:**
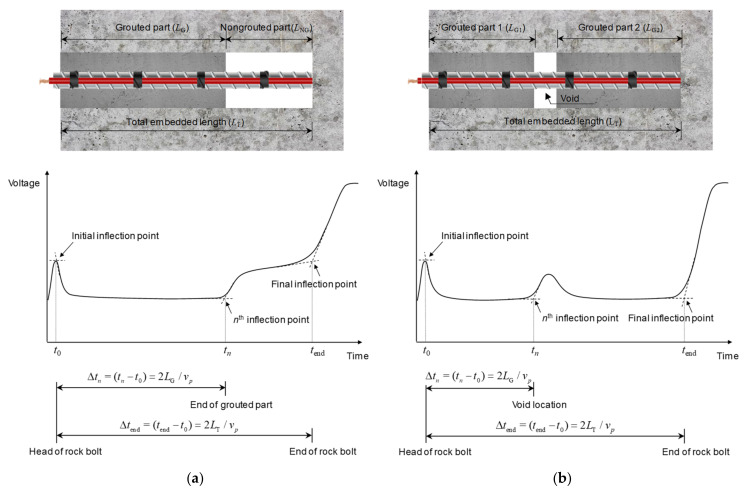
Waveforms of electromagnetic waves in defective rock bolts with: (**a**) non-grouted part at the end; (**b**) void.

In this study, an initial inflection point was determined as the intersection between the horizontal and negatively sloped tangent lines at the trace’s local maximum, in accordance with the tangent method, as shown in Figure 5. The nth inflection point, caused by a defect, was determined by locating the intersection between the horizontally flat and positively sloped tangent lines at the trace’s local minimum, in accordance with the tangent method, as shown in Figure 5. If a signal reflected at the end appears as two sloped lines, the sloped line method was adopted to identify the final inflection point. The final inflection point was determined by locating the intersection between two positively sloped tangent lines at the trace’s local minimum, as shown in Figure 5a. If a signal reflected at the end appears as a horizontally flat and a sloped line, the final inflection point was determined according to the tangent method. The intersection between the horizontally flat and positively sloped tangent lines at the trace’s local minimum was determined as the final inflection point, as shown in Figure 5b.

The round-trip travel time of electromagnetic waves reflected from the end of the rock bolt was calculated based on the time difference (Δ*t*_end_) between *t*_0_ (time at the initial inflection point) and *t*_end_ (time at the final inflection point). The round-trip travel time of electromagnetic waves reflected from defects was determined by the time difference (Δ*t_n_*) between *t*_0_ and *t_n_* (time at the *n*_th_ inflection point). The velocity of electromagnetic waves (*v_p_*) in the rock bolt was calculated by the ratio of twice the length of the rock bolt to the travel time (∆*t*_end_).

## 5. Experimental Program

Experimental studies were conducted to investigate the suitability of the smart sensing system using electromagnetic waves to inspect defects in rock bolts. Rock bolt specimens were prepared in two different conditions, namely non-embedded and embedded. The non-embedded condition indicates that rock bolts are only surrounded by cement grout, whereas they are not installed in the concrete block. The embedded condition indicates that non-embedded rock bolts are embedded in the concrete block to simulate rock bolts installed in rock mass. Electromagnetic wave velocities in non-embedded rock bolts were compared to those in embedded rock bolts to investigate the effect of the surrounding material. Further, non-grouted ratios and defect locations in rock bolts were evaluated using the electromagnetic wave velocity.

### 5.1. Construction of Rock Bolt Specimens

**Figure 6:**
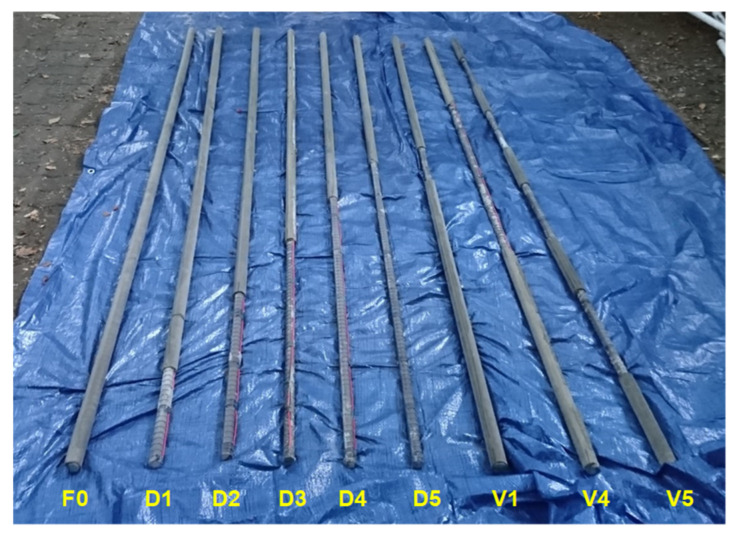
Rock bolt specimens for experiments in non-embedded condition.

**Figure 7:**
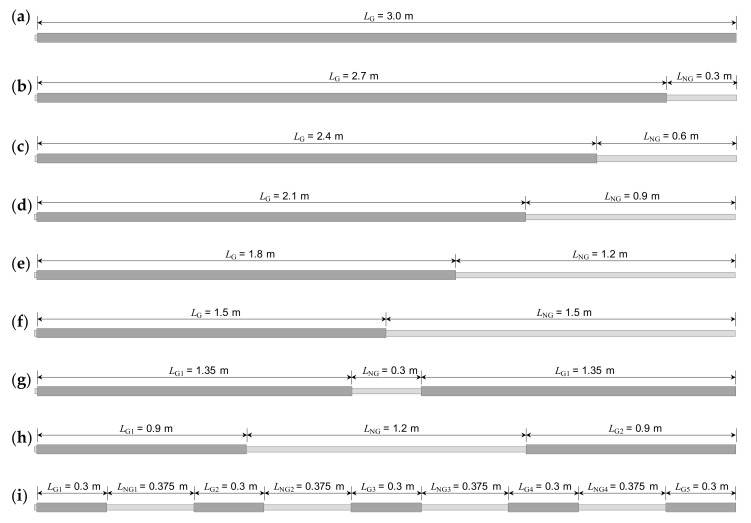
Schematic diagram of rock bolt specimens in non-embedded condition: (**a**) F0 (NGR = 0%); (**b**) D1 (NGR = 10%); (**c**) D2 (NGR = 20%); (**d**) D3 (NGR = 30%); (**e**) D4 (NGR = 40%); (**f**) D5 (NGR = 50%); (**g**) V1 (NGR = 10%); (**h**) V4 (NGR = 40%); (**i**) V5 (NGR = 50%). NGR: non-grouted ratio.

In total, nine rock bolt specimens were prepared in non-embedded condition, as shown in Figure 6. One was a fully grouted rock bolt, and the other eight were defective rock bolts. The fully grouted rock bolt was manufactured as follows: a rebar, in which the transmission line was already configured as described in Section 3.1, was inserted into a PVC pipe; the rebar was then suspended using nylon threads to be positioned at the center of the PVC pipe; both ends of the PVC pipe were capped with PVC caps to form an end seal; grout material was poured into the PVC pipe through rectangular holes on the pipe surface; a length of 10 mm of the head part of the rebar was not grouted to allow connection to the coaxial cable; the PVC pipe was removed after curing for 28 days. The internal diameter and length of the PVC pipe were 40.0 mm and 3.0 m, respectively. Consequently, the fully grouted rock bolt (F0) was 3.01 m in length, and its grouted length and diameter were 3.0 m and 40 mm, respectively, as shown in Figure 7a. The grout material constituted cement paste, which was a mixture of cement and water at a weight ratio of 1: 0.5 (w/c = 50%). The dielectric constant of the grout at 28-day curing was about 4.5.

The eight defective rock bolts were manufactured by partially grouting rock bolts, as shown in Figure 7b–i. During manufacturing, only the grouted parts of the rock bolts were covered with PVC pipes to be filled with the cement grout, while non-grouted parts were left uncovered. The non-grouted parts were therefore idealized as cylinder-shaped defects, and they were axisymmetric to the rebar as a simplification of the defective rock bolt model.

Five out of eight defective rock bolts had non-grouted parts at the ends, which simulate defects resulting from the outflow of grout due to gravity. Their respective non-grouted lengths were 0.3, 0.6, 0.9, 1.2, and 1.5 m, as shown in Figure 7b–f, respectively. The defective rock bolts shown in Figure 7b–f were numbered from D1 to D5, respectively.

The other three defective rock bolts had three different types of voids to simulate defective rock bolts resulting from unexpected factors, such as the collapse of the wall, ground water flow, and poor consistency of grout materials. One had a small void of 0.3 m length in the middle, as shown in Figure 7g. Another had a larger void of 1.2 m length in the middle, as shown in Figure 7h. The other had four voids of 0.375 m length at intervals of 0.3 m, as shown in Figure 7i, whose sum amounted to 1.5 m. The three defective rock bolts with voids shown in Figure 7g–i were numbered V1, V4, and V5, respectively.

**Figure 8:**
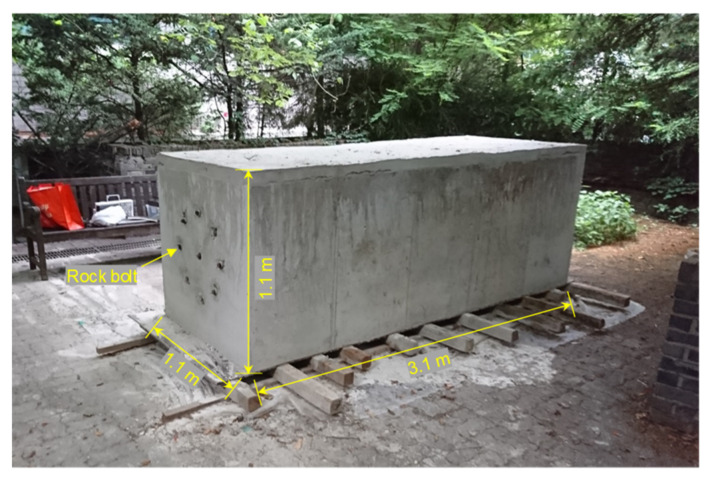
Concrete block with installed rock bolts for simulation of rock mass.

**Figure 9:**
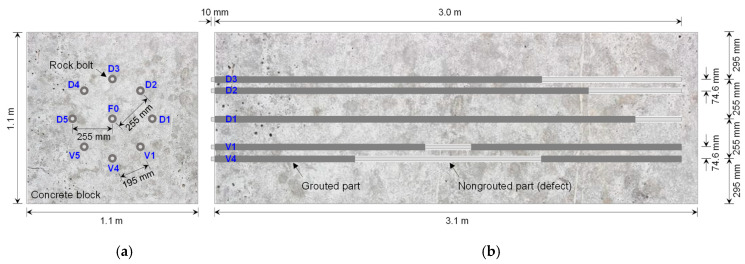
Diagram of concrete block with installed rock bolts: (**a**) front view; (**b**) side view.

The single fully grouted rock bolt and eight defective rock bolts in the non-embedded condition were finally embedded in a concrete block (see Figure 8) to simulate rock bolts installed in rock mass. Non-grouted parts were covered with PVC pipes to prevent them from being filled with concrete. Therein, a cuboid mold to cast a concrete block was constructed with steel panels (euro-form); the fully grouted rock bolt was then suspended at the center of the mold using nylon ropes; in the same manner, the eight defective rock bolts were arranged in a circular shape at equal intervals around the fully grouted rock bolt; concrete was then poured into the mold, and the mold was removed after curing for 28 days. The distance between the fully grouted rock bolt and defective rock bolts was set to be 255 mm, as shown in Figure 9a. The interval between defective rock bolts was set at approximately 195 mm. The embedded length of 3.01 m long rock bolts installed in the concrete block was 3.0 m, while the 10 mm length for the connection of the coaxial cable at the heads of rock bolts was exposed to air, as shown in Figure 9b. The length, width, and height of the concrete block were 3.1, 1.1, and 1.1 m, respectively. The compressive strength and dielectric constant of the concrete at 28-day curing were approximately 35 MPa and 4.2, respectively. The effects of PVC pipes on the propagation of electromagnetic waves were minor because the dielectric constant of the PVC pipe (*ε_r_* ≈ 4) is similar to that of concrete (*ε_r_* ≈ 4.2) [43].

In this study, the severity of the defect in the rock bolt was expressed by the non-grouted ratio. The non-grouted ratio (NGR) was defined as the ratio of the sum of the length of non-grouted parts (*L*_NG_) to the total length of the rock bolt (*L*_T_):

(18) $$\mathrm{Nongrouted}\;\mathrm{Ratio}\;(\%)=\frac{\mathrm{Sum}\;\mathrm{of}\;\mathrm{lengths}\;\mathrm{of}\;\mathrm{nongrouted}\;\mathrm{parts},\;L_{\mathrm{NG}}}{\mathrm{Total}\;\mathrm{length}\;\mathrm{of}\;\mathrm{rock}\;\mathrm{bolt},\;L_{T}}\times 100$$

According to Equation (18), a NGR of 0% denotes the fully grouted rock bolt. Further, the NGR of 50% indicates a half-grouted rock bolt. The NGR of 100% indicates the rebar.

### 5.2. Experimental Results

#### 5.2.1. Non-embedded Rock Bolts

**Figure 10:**
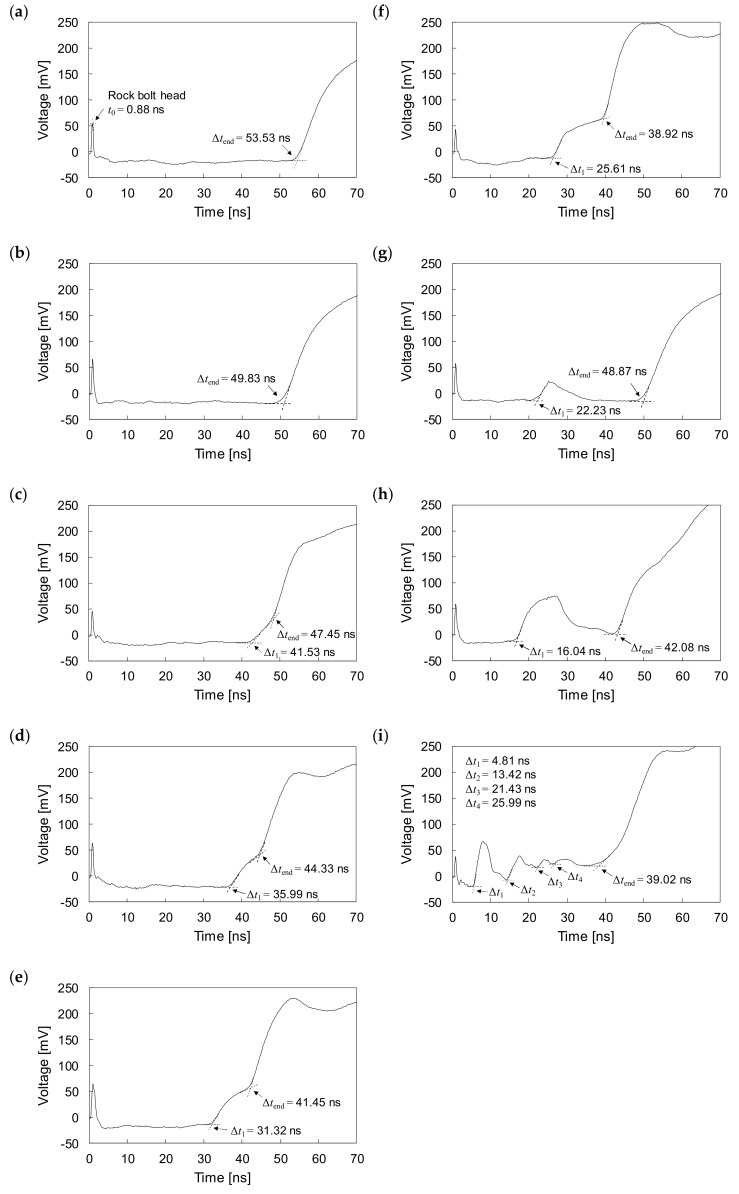
Measured signals in non-embedded rock bolts: (**a**) F0 (NGR = 0%); (**b**) D1 (NGR = 10%); (**c**) D2 (NGR = 20%); (**d**) D3 (NGR = 30%); (**e**) D4 (NGR = 40%); (**f**) D5 (NGR = 50%); (**g**) V1 (NGR = 10%); (**h**) V4 (NGR = 40%); (**i**) V5 (NGR = 50%).

The experiments were conducted with one fully grouted rock bolt and eight defective rock bolts under non-embedded conditions. The measured signal for the fully grouted rock bolt in the non-embedded condition is plotted in Figure 10a. The measured signals for defective rock bolts are plotted in Figure 10b–i. They show that electromagnetic waves are reflected at the head, end, and at defects of rock bolts. All round-trip travel times (*t*_0_) of electromagnetic waves reflected at the heads of all rock bolts in the non-embedded condition are 0.88 ns.

For the fully grouted rock bolt (F0, NGR = 0%), the electromagnetic wave reflections only appear at the head and at the end, as shown in Figure 10a. The amplitude of the signal decreases in the grouted part and then increases at the end of the fully grouted rock bolt. The time difference (Δ*t*_end_) between the round-trip travel times of electromagnetic waves reflected at the head and at the end is 53.53 ns.

For the defective rock bolts with non-grouted parts at the ends in the non-embedded condition, the reflections of electromagnetic waves are observed at the interfaces between the grouted and non-grouted parts, as well as at heads and at ends, as shown in Figure 10c–f. The signal amplitudes in the non-grouted parts are greater than those in the grouted parts. The time differences (Δ*t*_1_) between round-trip travel times of electromagnetic waves reflected at the heads and defects (i.e., interfaces between grouted parts and non-grouted parts) are 41.53, 35.99, 31.32, and 25.61 ns for D2 (NGR = 20%), D3 (NGR = 30%), D4 (NGR = 40%), and D5 (NGR = 50%), respectively. The Δ*t*_1_ decreases with increasing NGR. No reflection is observed at the defect for D1 (NGR = 10%), as the reflections at the defect and at the end overlap. The time differences (Δ*t*_end_) between round-trip travel times of electromagnetic waves reflected at the heads and ends are 49.83, 47.45, 44.33, 41.45, and 38.92 ns for D1, D2, D3, D4, and D5, respectively. The Δ*t*_end_ decreases with increasing NGR.

The reflections of electromagnetic waves at the borders of voids in defective rock bolts in the non-embedded condition are clearly detected, even in the presence of multiple voids, as shown in Figure 10g–i. The amplitudes of the signals increase in the voids, whereas they decrease in the grouted parts. For the defective rock bolt V1 (NGR = 10%), with one void in the middle, the time difference (Δ*t*_1_) between the round-trip travel times of electromagnetic waves reflected at the head and at the void is 22.23 ns, as shown in Figure 10g. For the defective rock bolt V4 (NGR = 40%) as shown in Figure 10h, the width of the reflected signal at the void is greater than in to V1, as the void in V4 is larger than that in V1. The Δ*t*_1_ of V4 is measured to be 16.04 ns. In the case of the defective rock bolt V5 (NGR = 50%), where four voids are present, four reflected signals are observed, as shown in Figure 10i. However, the peak amplitudes of the reflected signals at voids gradually decrease as the number of reflections increases. For the first, second, third, and fourth reflections, the Δ*t*_1_, Δ*t*_2_, Δ*t*_3_, and Δ*t*_4_ were measured at 4.81, 13.42, 21.43, and 25.99 ns, respectively. The time differences (Δ*t*_end_) between round-trip travel times of electromagnetic waves reflected at the heads and at the ends are 49.26, 42.08, and 39.02 ns for V1, V4, and V5, respectively. The Δ*t*_end_ of V1, V4, and V5 in the non-embedded condition are almost the same to those of D1, D4, and D5 in the non-embedded condition, respectively. The non-grouted ratios of V1, V4, and V5 are the same as those of D1, D4, and D5, respectively.

#### 5.2.2. Rock Bolts Embedded in Concrete Block

**Figure 11:**
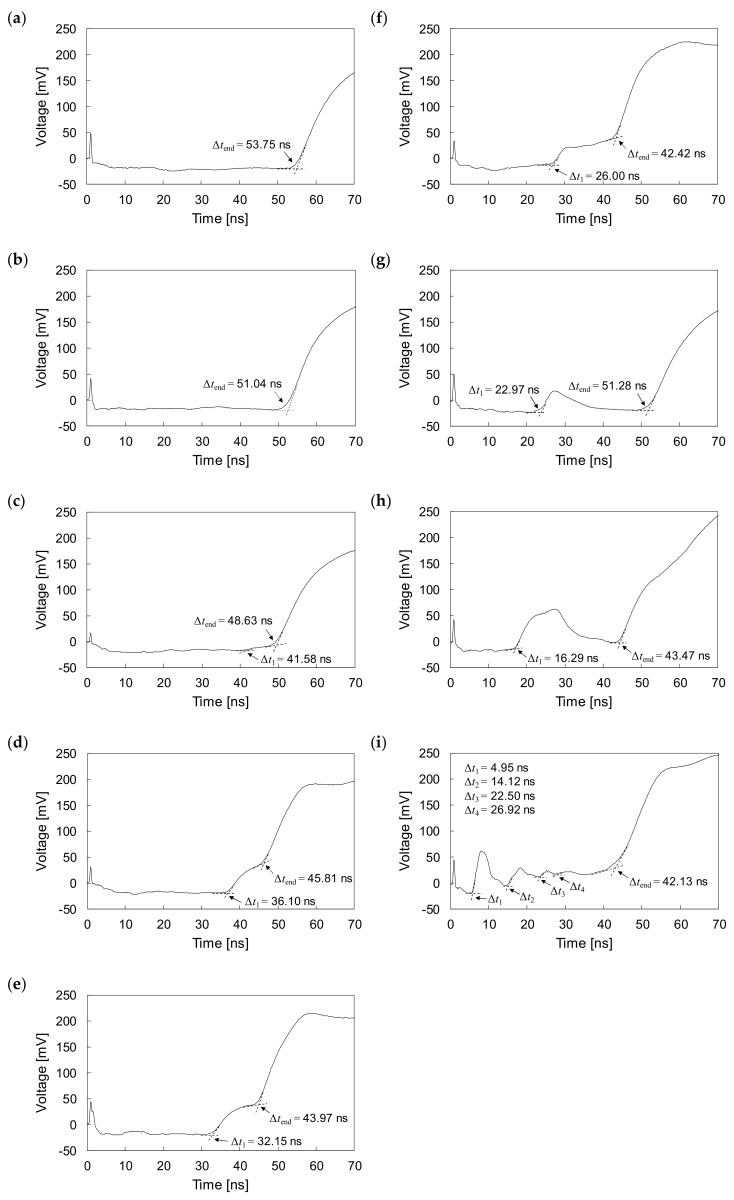
Measured signals in embedded condition for rock bolts: (**a**) F0 (NGR = 0%); (**b**) D1 (NGR = 10%); (**c**) D2 (NGR = 20%); (**d**) D3 (NGR = 30%); (**e**) D4 (NGR = 40%); (**f**) D5 (NGR = 50%); **(g)** V1 (NGR = 10%); (**h**) V4 (NGR = 40%); (**i**) V5 (NGR = 50%).

The experiments were performed with one fully grouted and eight defective rock bolts, which were embedded in the concrete block to simulate rock bolts installed in a rock mass. The measured signals for rock bolts in the embedded condition are plotted in Figure 11. The reflections of electromagnetic waves at the heads, ends, and at defects are clearly detected, even though rock bolts are embedded in the concrete block. The round-trip travel times (*t*_0_) of electromagnetic waves reflected at the heads of all rock bolts in the embedded condition are measured to be 0.88 ns.

For the fully grouted rock bolt (F0, NGR = 0%) in embedded condition, the electromagnetic waves are reflected at the head and end, as shown in Figure 11a. The amplitude of the signal is attenuated in the grouted part and increases at the end. The time difference (Δ*t*_end_) between round-trip travel times of electromagnetic waves reflected at the head and end of the fully grouted rock bolt is measured as 53.75 ns, which is slightly longer than the Δ*t*_end_ of the fully grouted rock bolt in the non-embedded condition.

For the defective rock bolts D2 (NGR = 20%), D3 (NGR = 30%), D4 (NGR = 40%), and D5 (NGR = 50%) with non-grouted parts at their ends in the embedded condition, the reflections of electromagnetic waves appear at the defects (i.e., interfaces between grouted and non-grouted parts) as well as at the heads and ends, as shown in Figure 11c–f, respectively. The amplitudes of measured signals are attenuated in the grouted parts, whereas they increase in the non-grouted parts. In the case of the defective rock bolt D1 (NGR = 10%), however, no reflection at the defect is observed, as reflections at the defect and the end overlap each other. For the defective rock bolts D2, D3, D4, and D5, the respective time differences (Δ*t*_1_) between round-trip travel times of electromagnetic waves reflected at the interface between grouted and non-grouted parts (i.e., defects) are 41.58, 36.10, 32.15, and 26.00 ns, as shown in Figure 11c–f, respectively. The time differences (Δ*t*_end_) between round-trip travel times of electromagnetic waves reflected at the heads and ends are 51.04, 48.63, 45.81, 43.97, and 42.42 ns for D1, D2, D3, D4, and D5, respectively. The Δ*t*_1_ and Δ*t*_end_ decrease with increasing NGR as in the case of non-embedded rock bolts. However, both Δ*t*_1_ and Δ*t*_end_ of embedded rock bolts are longer than those of non-embedded rock bolts.

The presence of voids in defective rock bolts in the embedded condition is clearly detected. The electromagnetic waves are observed to be reflected at voids, heads and ends of the defective rock bolts, as shown in Figure 11g–i. The signal amplitudes increase in the voids, whereas they decrease in the grouted parts. For the defective rock bolt V1 (NGR = 10%), with one void in the middle, the difference in the round-trip travel time (Δ*t*_1_) between electromagnetic waves reflected at the head and the void is 22.97 ns, as shown in Figure 11g. The Δ*t*_1_ of V1 in the embedded condition is longer than that in the non-embedded condition. For the defective rock bolt V4 (NGR = 40%), the Δ*t*_1_ is measured to be 16.29 ns, which is longer than that in the non-embedded condition, as shown in Figure 11h. The width of the reflected signal for V4 is larger than that for V1, as V4 has a larger void compared to V1. For the defective rock bolt V5 (NGR = 50%), where four voids are present, four reflections by voids appear in the measured signal, as shown in Figure 11i. The Δ*t*_1_, Δ*t*_2_, Δ*t*_3_, and Δ*t*_4_ for the first, second, third, and fourth reflections are measured to be 4.95, 14.12, 22.50, and 26.92 ns, respectively. The respective Δ*t*_1_, Δ*t*_2_, Δ*t*_3_, and Δ*t*_4_ of V5 in the embedded condition are longer than those in the non-embedded condition. The differences in the round-trip travel times (Δ*t*_end_) between electromagnetic waves reflected at the heads and ends for the V1, V4, and V5 are 51.28, 43.47, and 42.13 ns, respectively. The respective Δ*t*_end_ for V1, V4, and V5 are similar to the Δ*t*_end_ for D10, D40, and D50, of which non-grouted ratios are the same as non-grouted ratios of V1, V4, and V5, respectively. However, the respective Δ*t*_end_ for V1, V4, and V5 in the embedded condition are longer than those in the non-embedded condition.

## 6. Discussion and Analyses

### 6.1. Waveform of Measured Signals

In the cases of defective rock bolts with non-grouted parts at the ends, signal reflections at the interfaces between grouted and non-grouted parts appear with the same sign as the input signal. The propagation of electromagnetic waves is affected by the dielectric constant of air in non-grouted parts (i.e., defect), whereas in grouted parts it is affected by the dielectric constant of the cement grout. Notably, the dielectric constant of air (*ε_r_* ≈ 1) is smaller than that of the cement grout (*ε_r_* ≈ 4.5). The electrical impedance is inversely proportional to the dielectric constant, as described in Equation (17). The electrical impedance in the grouted parts is therefore smaller than that in the non-grouted parts. Thus, signals reflected from non-grouted parts, where the electrical impedance is larger than in the grouted parts, appear with the same sign as the input signal, as described in Equation (16). In cases of defective rock bolts with voids, voids generate two neighboring discontinuities on rock bolts: (1) the beginning of the void; (2) the end of the void. Thus, the reflections of the signals appear with the same phase at the beginnings of the voids, while they appear with the opposite phase at the ends of the voids.

The input signal used in the study is a step pulse, as shown in Figure 3, whereas measured signals are distorted as shown in Figure 10 and Figure 11, which show that reflections of measured signals appear with a degradation of rise time. The degradation of the rise time is caused by attenuation and dispersion, as described in Section 4.1. The cement grout and concrete are lossy medium that absorbs electrical energy as a consequence of its electrical conductivity, resulting in signal attenuation and dispersion. The conductivities of cement grout and concrete generally range from approximately 0.001 to 0.09 S/m, which is significantly larger than the conductivity of air, which ranges from 3 × 10^−15^ to 8 × 10^−15^ S/m [44,45,46]. Thus, electromagnetic waves propagating along the rebar are attenuated and dispersive due to surrounding cement grout and concrete, and the signals are consequently distorted. 

The rise time of the input signal is directly related to the spatial resolution of the measurement system. The length of the minimum resolved feature size (*l*) is given as [47]:

(19) $$l=\frac{t_{r}v_{c}}{2\sqrt{\varepsilon_{eff}}}$$

where *t_r_* is the rise time of the input step signal; *v_c_* is the velocity of electromagnetic waves in vacuum (3 × 10^8^ m/s); *ε_eff_* is the effective dielectric constant. The rise time of the input step signal generated by the TDR was 200 ps, and the dielectric constants of the grout and concrete were 4.5 and 4.2, respectively. Thus, *l* is estimated to be within the range of approximately 1.4 to 1.5 cm. However, degradation of the rise time due to signal distortion increases the rise time and consequently adversely affects the spatial resolution of the measurement system.

### 6.2. Velocity of Electromagnetic Waves

#### 6.2.1. Non-embedded Rock Bolts

**Figure 12:**
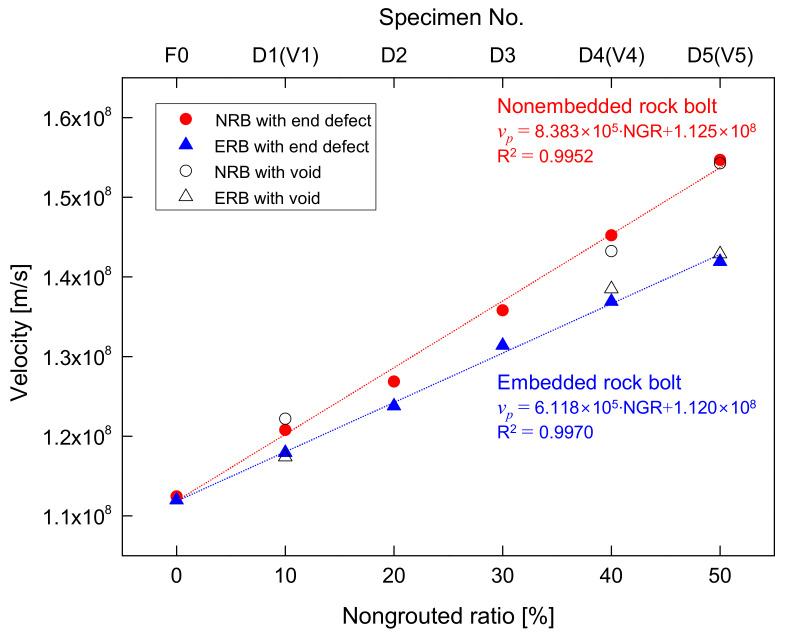
Comparison of velocities between non-embedded rock bolts (NRB) and embedded rock bolts (ERB) according to NGR.

The electromagnetic wave velocity in rock bolts, which depicts the ratio of round-trip travel distance to time difference (Δ*t*_end_) between *t*_0_ and *t*_end_, is summarized for all non-embedded rock bolts in Table 1 and plotted in Figure 12. The velocity of the electromagnetic waves in the fully grouted rock bolt (F0) in the non-embedded condition is 1.125 × 10^8^ m/s. In the cases of the defective rock bolts with non-grouted parts at the ends, the velocities of electromagnetic waves in D1, D2, D3, D4, and D5 are 1.208 × 10^8^, 1.269 × 10^8^, 1.358 × 10^8^, 1.452 × 10^8^, and 1.547 × 10^8^ m/s, respectively. The velocity in F0 is lower than that in the rebar (*v_p_* = 2.649 × 10^8^ m/s). Furthermore, the velocities in defective rock bolts with non-grouted parts at ends are greater than the velocity in F0. The velocity increases linearly with an increase in the NGR, as shown in Figure 12. The coefficient of determination (*R^2^*) for the relationship between the velocity and the non-grouted ratio is 0.9952. This indicates that the velocity can be used to evaluate non-grouted ratios with high reliability.

The electromagnetic wave velocity is inversely proportional to the dielectric constant of surrounding materials, as described in Equation (15), and dependent on the NGR. Hence, the velocity of electromagnetic waves propagating along the rebar depends on the dielectric constant of air, whereas the velocity of electromagnetic wave propagating along the fully grouted rock bolt depends on the dielectric constant of the cement grout. The dielectric constant of air (*ε_r_* ≈ 1) is smaller than that of the cement grout (*ε_r_* ≈ 4.5). In defective rock bolts, electromagnetic wave velocities are affected by both the dielectric constants of the cement grout and air at the grouted and non-grouted parts, respectively. The electromagnetic waves propagate according to their velocity in the rebar in the non-grouted part and according to their velocity in the rebar-cement grout mixture in the grouted part. Thus, the larger the NGR, the greater the velocity.

For the defective rock bolts with voids in the non-embedded condition, the velocities in V1, V4, and V5 are 1.222 × 10^8^ m/s, 1.432 × 10^8^ m/s, and 1.543 × 10^8^ m/s, respectively (see Table 1 and Figure 12). The respective velocities in V1, V4, and V5 are similar to those in D1, D4, and D5, as non-grouted ratios of V1, V4, and V5 are the same as those of D1, D4, and D5, respectively. This suggests that electromagnetic waves propagate according to the velocity of the rebar in the non-grouted part and according to the velocity of the rebar-cement grout mixture in the grouted part. Hence, the severity of defects in rock bolts may be evaluated by the relationship between the electromagnetic wave velocity and NGR, regardless of defect locations.

#### 6.2.2. Rock Bolts Embedded in Concrete Block

The velocities of electromagnetic waves reflected at ends of rock bolts in the embedded condition are summarized in Table 1 and plotted in Figure 12. The velocity of electromagnetic waves in the fully grouted rock bolt (F0) in embedded condition is 1.120 × 10^8^ m/s. For the defective rock bolts with non-grouted parts at their ends, the velocities of electromagnetic waves in D1, D2, D3, D4, and D5 are 1.179 × 10^8^, 1.238 × 10^8^, 1.314 × 10^8^, 1.369 × 10^8^, and 1.419 × 10^8^ m/s, respectively. Figure 12 shows that the velocity of electromagnetic waves in rock bolts in the embedded condition linearly increases with an increase in the NGR. Furthermore, the linear relationship between the velocity and the non-grouted ratio is 0.9970(*R^2^*). This demonstrates that the velocity is a reliable indicator for evaluating the non-grouted ratio of rock bolts. However, the velocities in rock bolts in the embedded condition are lower than those in the non-embedded condition. Furthermore, a larger NGR indicates a greater difference in the velocity between rock bolts in non-embedded and embedded conditions.

The velocity in grouted parts of rock bolts is only affected by the dielectric constant of the cement grout in the non-embedded condition, whereas it is affected by both dielectric constants of the cement grout and concrete in the embedded condition. However, the dielectric constant of cement grout (*ε_r_* ≈ 4.5) is slightly larger than that of concrete (*ε_r_* ≈ 4.2). The velocity in F0 in the embedded condition is therefore slightly lower than that in the non-embedded condition. For defective rock bolts with non-grouted parts at their ends, the velocity in non-grouted parts is only affected by air in the non-embedded condition, whereas it is affected by both air and concrete in the embedded condition. The velocity in the non-grouted part in the embedded condition is calculated to be 2.570 × 10^8^ m/s, which is lower than the velocity in the rebar (*v_p_* = 2.649 × 10^8^ m/s), by estimating the difference in the velocity between the fully grouted and the defective rock bolt based on the relationship shown in Figure 12. Thus, the difference in the velocity between rock bolts in non-embedded and embedded conditions increases with increasing NGR, as the dielectric constant of concrete is significantly larger than that of air.

For defective rock bolts with voids in the embedded condition, the velocities in V1, V4, and V5 are 1.174 × 10^8^, 1.385 × 10^8^, and 1.429 × 10^8^ m/s, respectively. The respective velocities in V1, V4, and V5 are almost the same as those in D1, D4, and D5, as they have the same NGR. The velocities in V1, V4, and V5 in the embedded condition are lower than those in the non-embedded condition. This is because velocities of electromagnetic waves in voids are affected by both the dielectric constant of air and that of concrete in the embedded condition.

### 6.3. Estimation of Defect Locations

#### 6.3.1. Non-Embedded Rock Bolts

A defect location is estimated as half the product of the velocity in the grouted part (*v*’*_p_*) and the difference in the round-trip travel time (Δ*t_n_*) between electromagnetic waves reflected at the head and at the defect, as described in Figure 5. Therefore, the defect location is the location of the beginning of the defect, which is the interface between the grouted and non-grouted parts. The velocity (*v_p_*) in rock bolts in the non-embedded condition is calculated according to the relationship between the velocity and NGR from Figure 12, as follows:

(20) $$v_{p}\;(m/s)=8.383\times 10^{5}\cdot \mathrm{NGR}\;(\%)+1.125\times 10^{8}$$

In accordance with Equation (20), the *v_p_* for the fully grouted rock bolt (NGR = 0%) of 3.01 m length in the non-embedded condition is calculated to be 1.125 × 10^8^ m/s. Δ*t*_end_ denotes the ratio of twice the length of the rock bolt to *v_p_*. Thus, Δ*t*_end_ is calculated to be 53.51 ns. The travel time for the exposed part (10 mm length) in the rebar is 75.49 ps based on the velocity in the rebar (*v_p_* = 2.649 × 10^8^ m/s). Thus, the travel time for the grouted part of 3 m length is 53.44 ns, which is the difference between 75.49 ps and 53.51 ns, and the velocity (*v*’*_p_*) is 1.123 × 10^8^ m/s. Considering that Δ*t*_1_ for defective rock bolt D2 with the non-grouted part at the end is 41.53 ns, the defect location for D2 is obtained as follows:

(21) $$\begin{array}{ll} \mathrm{Defect}\;\mathrm{location}\;(m) & =0.5\times {v^{\prime }}_{p}\times \Delta t_{1}+\mathrm{exposed}\;\mathrm{rebar}\;\mathrm{length}\\ & =0.5\times 1.123\times 10^{8}m/s\times 41.53\;\mathrm{ns}+10\;\mathrm{mm}\\ & =2.342\;m \end{array}$$

where 0.5 × *v*’*_p_*× Δ*t*_1_ corresponds to the length of the grouted part in D2. In the same manner, the defect locations of defective rock bolts D3, D4, and D5 with non-grouted parts at ends are calculated considering that the values of Δ*t*_1_ for D3, D4, and D5 are 35.99, 31.32, and 25.61 ns, respectively, as denoted in Figure 10 and summarized in Table 2. Therefore, the calculated defect location for D3 is 2.031 m, which is similar to the actual location of 2.110 m, as listed in Table 2. The estimated defect locations in D4 and D5 are 1.769 m and 1.448 m, respectively, which are in good agreement with the actual respective locations of 1.810 m and 1.510 m (Table 2).

To estimate defect locations of defective rock bolts with voids (V1, V4, and V5), electromagnetic waves are considered to propagate with the velocity *v_p_* along the rebar (*v_p_* = 2.649 × 10^8^ m/s) in non-grouted parts (i.e., voids). For V1 and V4, Δ*t*_1_ at their first reflections are 22.23 ns and 16.04 ns, respectively, as denoted in Figure 10 and summarized in Table 2. The respective defect locations in V1 and V4 are therefore estimated to be 1.258 m and 0.910 m, which are similar to the actual locations of 1.360 m and 0.910 m, respectively, as represented in Table 2. The respective Δ*t*_1_, Δ*t*_2_, Δ*t*_3_, and Δ*t*_4_ for the first, second, third, and fourth reflections in V5 are 4.81, 13.42, 21.43, and 25.99 ns, respectively (see Figure 10 and Table 2). Thus, the respective first, second, third, and fourth defect locations are estimated to be at 0.280, 0.980, 1.645, and 2.118 m, which are in good agreement with the actual defect locations of 0.310, 0.985, 1.660, and 2.335 m, respectively (see Table 2).

The estimated defect locations for defective rock bolts in the non-embedded condition are in good agreement with the actual defect locations. Errors in defect location estimation range from 0.03% to 9.75%, as listed in Table 2.

#### 6.3.2. Rock Bolts Embedded in Concrete Block

The velocity (*v_p_*) used for the estimation of defect locations in rock bolts in the embedded condition from Figure 12 is:

(22) $$v_{p}\;(m/s)=6.118\times 10^{5}\cdot \mathrm{NGR}\;(\%)+1.120\times 10^{8}$$

For the fully grouted rock bolt (NGR = 0%) of 3.01 m length in the embedded condition, the *v_p_* is calculated to be 1.120 × 10^8^ m/s and Δ*t*_end_ is therefore 53.75 ns. Considering the travel time and the velocity for the exposed rebar, the travel time and the velocity (*v*’*_p_*) for the grouted part of 3 m length are 53.67 ns and 1.118 × 10^8^ m/s, respectively. In accordance with Equation (21), the defect location for the defective rock bolt D2 with the non-grouted part at the end is estimated to be 23.334 m by taking Δ*t*_1_ = 41.58 ns into account, which is similar to the actual location of 2.410 m. For defective rock bolts D3, D4, and D5 with non-grouted parts at ends, the respective Δ*t*_1_ are 36.10, 32.15, and 26.00 ns, as shown in Figure 11 and summarized in Table 3. The respective locations of defects in D3, D4, and D5 are therefore estimated to be 2.028, 1.807, and 1.463 m, which are in agreement with the actual locations of 2.110, 1.810, and 1.510 m, respectively (see Table 3).

The electromagnetic waves in non-grouted parts (i.e., voids) for the embedded condition propagate at the velocity of 2.570 × 10^8^ m/s, as explained in Section 6.2. Thus, the defect location in V1 is calculated to be 1.294 m, which is similar to the actual location of 1.360 m, as summarized in Table 3, considering that the Δ*t*_1_ is 22.97 ns (Figure 11g). The Δ*t*_1_ for V4 is 16.29 ns, such that the calculated location is 0.921 m, which is in agreement with the actual location of 0.910 m, as summarized in Table 3. In the case of V5, the respective Δ*t*_1_, Δ*t*_2_, Δ*t*_3_, and Δ*t*_4_ for the first, second, third, and fourth reflections are 4.95, 14.12, 22.50, and 26.92 ns, respectively (see Figure 11i and Table 3). The respective locations of first, second, and third defects are therefore calculated to be 0.286, 1.011, and 1.691 m, which are similar to the actual locations of 0.310, 0.985, and 1.660 m, respectively. The calculated location of the fourth defect is 2.150 m, which relatively less than the actual location of 2.335 m. This is because multiple reflections further distort signals, and thus the fourth reflection signal, which is significantly distorted, makes it difficult to determine the travel time.

The calculated defect locations for the defective rock bolts in the embedded condition show good agreement with the actual defect locations. Errors in the estimated defect locations are estimated to range from 0.16% to 7.90%, as listed in Table 3.

## 7. Summary and Conclusions

A smart sensing method using electromagnetic waves for inspecting defects in rock bolts was suggested in this study. Two-conductor transmission lines were configured in rock bolts by installing electrical wires alongside rebars to transport electrical signals. The generation and detection of electromagnetic waves were accomplished using the TDR. Experimental studies were performed with one fully grouted and eight defective rock bolts. The eight defective rock bolts consisted of five defective rock bolts with non-grouted parts at the ends and three defective rock bolts with voids. The five defective rock bolts with non-grouted parts at the ends were prepared with different non-grouted ratios of 10%, 20%, 30%, 40%, and 50%. Two out of three defective rock bolts with voids had one void with a NGR of 10% and 40%, respectively. The other one had four voids with a NGR of 50%. The experiments were carried out in non-embedded and embedded conditions.

The experimental results showed that electromagnetic waves were reflected at defects as well as at the head and end of rock bolts. In defective rock bolts with four voids, all four voids located on the same rock bolt were clearly detected. The reflections of electromagnetic waves at defects were caused by differences in the electrical impedance between grouted and non-grouted part. Because the electrical impedance is inversely proportional to a dielectric constant, and the dielectric constant of air is smaller than that of the cement paste, the electrical impedance in defects is greater than that in the grouted parts. Thus, the reflected signals at defects appear with the same phase to the input signal. The velocities of electromagnetic waves propagating along the fully grouted rock bolt were lower than those traveling along the defective rock bolts. Further, the velocity of electromagnetic waves increased at higher non-grouted ratios. However, the velocities in rock bolts in the non-embedded condition were greater than those in embedded rock bolts. The electromagnetic wave velocity is affected by the dielectric constant of surrounding materials. The variations in velocity were therefore attributed to the differences in dielectric constants between air, grout, and concrete. Future studies can provide more information regarding the influence of the water-cement ratio of the grout on the velocity of electromagnetic waves in rock bolts. The locations of defects were estimated using the round-trip travel time and velocity of the electromagnetic waves. The results showed good agreement between estimated and actual defect locations. This study suggests that the smart sensing using electromagnetic waves may be an effective method to inspect defects in rock bolts.

The smart sensing system with the parallel two-conductor transmission line is realized using rock bolts. Thus, electrical wires should be installed alongside rock bolts prior to their installation. This method would allow permanent monitoring of rock bolts unless an electrical wire or rock bolt is damaged by rock movement or weathering. The parallel two-conductor transmission line is the simplest method to configure in rock bolts. However, further studies are required for a twisted pair and three-conductor transmission lines to improve signal sensitivity.

## Figures and Tables

**Table 1:** Round-trip travel times reflected at rock bolt ends (Δ*t*_end_) and propagation velocities (*v_p_*).

Specimen No	NGR (%)	*L*_T_ (m)	*L*_NG_ (m)	Non-Embedded		Embedded		Remarks
				Δ*t*_end_ (ns)	*v_p_* (10^8^ m/s)	Δ*t*_end_ (ns)	*v_p_* (10^8^ m/s)	
F0	0	3.0	0	53.53	1.125	53.75	1.120	Fully grouted rock bolt
D1	10	3.0	0.3	49.83	1.208	51.04	1.179	Defect at the end
D2	20	3.0	0.6	47.45	1.269	48.63	1.238	
D3	30	3.0	0.9	44.33	1.358	45.81	1.314	
D4	40	3.0	1.2	41.45	1.452	43.97	1.369	
D5	50	3.0	1.5	38.92	1.547	42.42	1.419	
V1	10	3.0	0.3	49.26	1.222	51.28	1.174	One void at the middle
V4	40	3.0	1.2	42.08	1.432	43.47	1.385	
V5	50	3.0	1.5	39.02	1.543	42.13	1.429	Four voids at intervals of 0.3 m

* Note that the *v_p_* of the rebar in air is 2.649 × 10^8^ m/s.

**Table 2:** Round-trip travel times reflected at defects (Δ*t_n_*) and estimated defect locations for defective rock bolts in non-embedded condition.

Specimen No	NGR (%)	Δ*t_n_* (ns)	Defect Location (m)		Error (%)	Remarks
			Estimated	Actual		
D1	10	-	-	2.710	-	Defect at the end
D2	20	41.53	2.342	2.410	2.84	
D3	30	35.99	2.031	2.110	3.76	
D4	40	31.32	1.769	1.810	2.29	
D5	50	25.61	1.448	1.510	4.13	
V1	10	22.23	1.258	1.360	7.50	One void at the middle
V4	40	16.04	0.910	0.910	0.03	
V5	50	4.81	0.280	0.310	9.75	Four voids at intervals of 0.3 m
		13.42	0.980	0.985	0.54	
		21.43	1.645	1.660	0.89	
		25.99	2.118	2.335	9.31	

**Table 3:** Round-trip travel times reflected at defects (Δ*t_n_*) and estimated defect locations for defective rock bolts in embedded condition.

Specimen No	NGR (%)	Δ*t_n_* (ns)	Defect Location (m)		Error (%)	Remarks
			Estimated	Actual		
D1	10	-	-	2.710	-	Defect at the end
D2	20	41.58	2.334	2.410	3.16	
D3	30	36.10	2.028	2.110	3.89	
D4	40	32.15	1.807	1.810	0.16	
D5	50	26.00	1.463	1.510	3.10	
V1	10	22.97	1.294	1.360	4.86	One void at the middle
V4	40	16.29	0.921	0.910	1.16	
V5	50	4.95	0.286	0.310	7.59	Four voids at intervals of 0.3 m
		14.12	1.011	0.985	2.64	
		22.50	1.691	1.660	1.89	
		26.92	2.150	2.335	7.90	
